# Assessment of anti-cyclic citrullinated peptide in psoriatic arthritis

**DOI:** 10.1186/1756-0500-2-44

**Published:** 2009-03-19

**Authors:** Nermeen SA Abdel Fattah, Hanan E Hassan, Zenab A Galal, El Sayed E El Okda

**Affiliations:** 1 Department of Dermatology and Venereology, Faculty of Medicine, Ain Shams University, Cairo, Egypt; 2 Department of Internal Medicine, Faculty of Medicine, Ain Shams University, Cairo, Egypt; 3 Department of Clinical Pathology, Faculty of Medicine, Ain Shams University, Cairo, Egypt; 4 Department of Community Medicine, Faculty of Medicine, Ain Shams University, Cairo, Egypt

## Abstract

**Background:**

Anti-cyclic citrullinated peptides (anti-CCP) are highly specific diagnostic and prognostic markers for rheumatoid arthritis (RA). They have been also found in psoriatic arthritis (PsA), with controversies as regards clinical and radiological associations. The current study assessed anti-CCP in PsA and determined its clinical and radiological associations.

**Methods:**

Four groups contributed to this study. 40 PsA, 40 psoriasis without arthritis, 40 RA and 40 healthy controls. They were tested for anti-CCP. Clinical and radiological data were collected and statistically compared between anti-CCP-positive and -negative PsA patients.

**Results:**

Seven PsA (17.5%) and 34 RA (85%) were seropositive for anti-CCP. Patients of other groups were anti-CCP-negative. Regarding anti-CCP concentrations, highly significant difference existed between different groups and between anti-CCP-positive and -negative PsA. Significantly higher numbers of involved, swollen and tender joints, deformities and functional impairment of peripheral joints and radiological changes were found in anti-CCP-positive PsA.

**Conclusion:**

Anti-CCP may be found in PsA and are associated with higher number of involved, swollen and tender joints, with deformities and functional impairment of peripheral joints and with erosive arthritis.

## Background

Psoriatic arthritis (PsA) is an inflammatory joint disease that shares features of rheumatoid arthritis (RA). This makes the differential diagnosis between PsA and RA in a patient with psoriasis difficult [1,2]. Moreover, laboratory test with specificity for PsA is still unavailable [1-3]. Various classifications and diagnostic criteria for PsA have been developed [4] of which the Moll and Wright [5] criteria remain the simplest and the most frequently used [4].

Antibodies targeting cyclic citrullinated peptide (anti-CCP) have been detected in the serum of RA patients, with high sensitivity and specificity [6-8] and serve as a powerful serologic marker for early diagnosis and prognostic prediction of joint destruction and disease progression [9-11]. Anti-CCP have been also identified in PsA with controversies as regards its clinical and radiological associations [12-18]. The present study aimed to assess anti-CCP in PsA and to determine its clinical and radiological associations.

## Methods

### Study design

This study represents a case-control study. Subjects were randomly selected from those attending the outpatient clinics of dermatology, rheumatology unit of internal medicine and rheumatology and rehabilitation departments of Ain Shams University Hospitals, Cairo, Egypt in the period from January 2006 to April 2008.

### Study population

Four groups contributed to this study. Group I included 40 PsA patients. PsA was diagnosed based on Moll and Wright criteria [5]. The presence of positive rheumatoid factor (RF) was not considered an exclusion criterion [19]. Group II included 40 psoriatic patients without arthritis. Group III included 40 RA patients diagnosed according to the American Rheumatism Association (ARA) 1987 revised criteria [20] and group IV included 40 healthy volunteers. Prior to initiation of the study, every subject was informed about the aim of the study and gave consent.

All PsA patients were then subjected to:

a-Full history taking.

b-Dermatological examination including examination of psoriatic nail changes and Psoriasis Area and Severity Index (PASI) score [21].

c-Peripheral and axial joint evaluation according to Moll and Wright criteria, modified by Helliwell et al [4]. Physical examination of joints included type and number of joints involved, range of movement, number of swollen and tender joints and presence of deformity or mutilating arthritis. Oligoarthritis was considered when <5 joints were involved and polyarthritis when ≥ 5 joints were involved. Symmetric arthritis considered when bilateral involvement >50% of affected joint was present.

d-Exclusion of RA using ARA 1987 revised criteria [20].

e-Plain radiographs of hands and feet and its evaluation with emphasis on the presence of erosions, ankylosis, cupping, whittling, deformities or destruction [4].

Blood sample was taken from all studied subjects. Serum was extracted and frozen at -70°C until assayed for anti-CCP and RF. Anti-CCP assay (cut-off, 5 U/ml) was done using second generation ELISA (Axis Shield, Dundee, UK). RF was assayed (cut-off, 15 IU/ml) using the commercial ELISA (Sigma Diagnostics, St Louis, MO, USA). HCV antibody was tested in RF-positive PsA patients using third generation ELISA (ETI-AB-HCVK4, DiaSorin S.p.A., 13040 Saluggia (Vercelli) – Italy).

### Statistical analysis

Statistical analysis was carried out using the statistical Package for the Social Sciences (SPSS) version 12. Quantitative data were presented as mean, standard deviation (SD), median and range while qualitative data were presented as number (n) and percentage (%). Differences between continuous data were tested using Mann-Whitney and Student's t-tests and differences between categorical data were tested using Chi-square and Fisher's exact tests. Analysis of variance (ANOVA) and Chi-square tests were used to compare more than 2 groups as regards quantitative and qualitative variables respectively. Post-hoc tests were used for multiple comparisons between groups. P-value ≤ 0.05 was considered statistically significant and ≤ 0.001 highly significant.

## Results

The study included 4 groups of studied subjects (table 1). The first group included 40 patients with PsA; 18 males and 22 females with mean age 42.85 ± 6 years. The second group included 40 patients with psoriasis without arthritis; 26 males and 14 females with mean age 43 ± 4 years. The third group included 40 patients with RA; 9 males and 31 females with mean age 46.9 ± 5 years. The fourth group included 40 healthy volunteers; 16 males and 24 females with mean age 40 ± 4 years. No statistically significant differences were found between different groups as regards their age (P = 0.13).

**Table 1:** Age and sex distribution of studied groups

	**PsA** **(n = 40)**	**Psoriasis** **without arthritis** **(n = 40)**	**RA** **(n = 40)**	**Healthy** **controls** (**n = 40)**
**Age (years)**				
Mean ± SD	42.85 ± 6	43 ± 4	46.9 ± 5	40 ± 4

**Sex**				
Males n(%)	18(45%)	26 (65%)	9 (22.5%)	16 (40%)
Females n(%)	22 (55%)	14 (35%)	31 (77.5%)	24 (60%)

PsA: psoriatic arthritis, RA: rheumatoid arthritis

### Results of anti-CCP and RF in studied subjects

**Figure 1:**
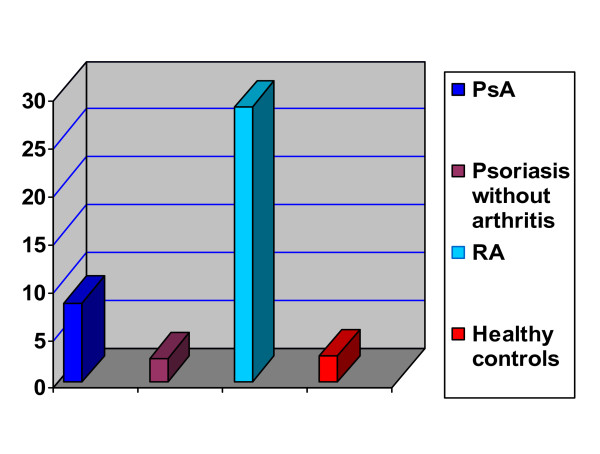
**Mean anti-CCP concentrations of studied groups**.

As regards anti-CCP, 7/40 PsA (17.5%) and 34/40 RA patients (85%) were seropositive with respect to the cut-off value. Patients with psoriasis without arthritis and healthy controls showed anti-CCP concentrations below cut-off value (anti-CCP-negative). On comparing the different groups together, statistically highly significant difference was found (table 2). As regards anti-CCP concentrations (Figure 1), statistically highly significant difference existed between the different studied groups (table 3) as well as between anti-CCP-positive PsA, anti-CCP-negative PsA, psoriasis without arthritis and healthy controls (table 4). Post-hoc test revealed significant differences between PsA and psoriasis without arthritis (P = 0.012) and between PsA and healthy controls (P = 0.010). Statistically highly significant differences were found between RA and PsA, between RA and psoriasis without arthritis, between RA and healthy controls and between anti-CCP-positive and -negative PsA patients (P = 0.001). On the other hand, no statistical difference was present between anti-CCP-positive PsA and RA (P = 0.1464), and between psoriasis without arthritis and healthy controls (0.848).

**Table 2:** Anti-CCP seropositivity of studied groups

	**PsA** **(n = 40)**	**Psoriasis** **without arthritis** **(n = 40)**	**RA** **(n = 40)**	**Healthy** **controls** (**n = 40)**	**P-value**
**Anti-CCP**					
**-negative **n (%)	33 (82.5%)	40 (100%)	6 (15%)	40 (100%)	**<0.001**
**-positive **n (%)	7 (17.5%)	0	34 (85%)	0	

PsA: psoriatic arthritis, RA: rheumatoid arthritis

**Table 3:** Anti-CCP concentrations in studied groups

	**PsA** **(n = 40)**	**Psoriasis** **without arthritis** **(n = 40)**	**RA** **(n = 40)**	**Healthy** **Controls** (**n = 40)**	**P-value**
**Mean ± SD**	8.25 ± 11.6	2.435 ± 1.3	28.63 ± 11.75	2.68 ± 1.3	**< 0.001**
**Median**	3.8	2.5	32	1.8	

PsA: psoriatic arthritis, RA: rheumatoid arthritis

**Table 4:** Anti-CCP concentrations in anti-CCP-positive and -negative PsA, psoriasis without arthritis and healthy controls

	**anti-CCP** **positive PsA** **(n = 7)**	**anti-CCP** **negative PsA** **(n = 33)**	**Psoriasis** **without arthritis** **(n = 40)**	**Healthy** **controls** (**n = 40)**	**P-value**
**Mean ± SD**	34.7 ± 8	3.21 ± 1.16	2.435 ± 1.3	2.68 ± 1.3	**0.001**

PsA: psoriatic arthritis, RA: rheumatoid arthritis

As regards RF, seropositivity was found in 4 PsA patients with respect to the cut-off value; 2 of them were also positive for anti-CCP. None of RF-positive PsA patients were HCV-positive. All RA patients were RF-seropositive; 34 of them showed anti-CCP seropositivity in addition. Two psoriatic patients without arthritis and two healthy controls were RF-positive. No statistical difference was found between anti-CCP-positive and -negative PsA patients as regards RF concentrations (P = 0.0714) (table 5).

**Table 5:** Characteristics of anti-CCP-positive and -negative PsA patients

	**Anti-CCP-positive** **(n = 7)**	**Anti-CCP-negative** **(n = 33)**	**P-value**
**Age (years**)			
Mean ± SD (range)	43 ± 5.7 (39–50)	47 ± 6.9 (33–54)	0.161*

**Sex**			
**Male **n (%)	3 (42.86%)	15 (45.45%)	
**female **n (%)	4 (57.14%)	18 (54.55%)	0.67**

**Disease Duration (months)**			
Mean ± SD (range)	40.7 ± 12.34 (30–54)	37 ± 9 (24–60)	0.36*

**PASI**			
Mean ± SD (range)	10 ± 1.53 (8–11)	8.86 ± 1.9 (7–13)	0.146*

**Psoriatic nail changes**			
n (%)	4 (57.14%)	29 (87.9%)	0.087**

**Involved joints**			
median (range)	15 (10–24)	6 (5–10)	0.0135*

**Swollen joints**			
median (range)	2.5 (2–7)	1 (0–2)	0.023*

**Tender joints**			
median (range)	9 (3–16)	3 (0–8)	0.009*

**Spondyloarthritis**			
n (%)	1 (14.28%)	5 (15.15%)	0.901**

**Oligoarthritis**			
n (%)	0	6 (18.18%)	0.567**

**Symmetric Polyarthritis**			
n (%)	7 (100%)	12 (36.36%)	0.003**

**Asymmetric Polyarthritis**			
n (%)	0	2 (6.06%)	0.542**

**Deformities of peripheral joints**			
n (%)	7 (100%)	15 (45.45%)	0.011**

**Functional impairment of peripheral joints**			
n (%)	7 (100%)	15 (45.45%)	0.011**

**Radiological changes:** **peripheral deformities/erosions**			
n (%)	7 (100%)	15 (45.45%)	0.011**

**RF concentrations (IU/ml)**			
Median (range)	15 (7–30)	9 (4–33)	0.0714*

**Anti-CCP concentrations (U/ml)** Mean ± SD (range)	34.7 ± 8 (30–45)	3.21 ± 1.16 (1–5)	0.0001*

* Student's t and Mann Whitney tests.

** Chi-square and Fisher's exact tests

### Characteristics of anti-CCP-positive and-negative PsA patients

The demographic, clinical, laboratory and radiological features of anti-CCP-positive and -negative PsA patients are summarized in table 5. All PsA patients had psoriasis vulgaris and arthritis. The arthritis was preceded by psoriasis in all patients, with mean ± SD: 22 ± 4 months. The age, sex, disease duration and PASI score of anti-CCP-positive PsA were statistically insignificant from anti-CCP-negative patients. Moreover, no statistical significance existed between both groups as regards number of patients with psoriatic nail changes, oligoarthritis or spondyloarthritis. All patients with PsA and positive anti-CCP had symmetric polyarthritis of peripheral small joints. One of them had spondyloarthritis in addition. Spondyloarthritis was more frequent in the anti-CCP-negative PsA patients (5/33). One patient had pure spondyloarthritis. In the other patients, spondyloarthritis was present in addition to oligoarthritis (2/5) or polyarthritis (2/5). None of the anti-CCP-positive or -negative patients had arthritis mutilans. Statistically significant higher numbers of involved, swollen and tender joints existed in anti-CCP-positive PsA patients. Moreover, anti-CCP-positive patients had deformities and functional impairment of peripheral joints, and radiological changes with statistically significant difference from anti-CCP-negative patients.

### Bones and joints characteristics of anti-CCP-positive PsA patients

All anti-CCP-positive PsA patients had symmetric polyarthritis of peripheral small joints, limited range of movement of interphalangeal joints and swan neck deformity of distal interphalangeal joints, evident by both clinical and radiological examination. They also showed peripheral boney erosions on radiographs. Ankylosis of different interphalangeal joints was detected in 2 patients.

## Discussion

In the present study, anti-CCP were found in 17.5% of PsA patients with significantly higher serum concentrations from psoriasis without arthritis and healthy controls and insignificant from RA. This adds further support to previous studies [12-18] concerning the detection of anti-CCP in PsA.

Significantly higher anti-CCP serum concentration, clinical and radiological features were found in anti-CCP-positive PsA patients when compared with anti-CCP-negative patients, suggesting the importance of anti-CCP testing in PsA. Statistically significant higher numbers of involved, swollen and tender joints were found in anti-CCP-positive patients. Symmetric polyarthritis, limited range of movement and deformities of small peripheral joints were also present in the anti-CCP-positive PsA patients with statistically significant differences from anti-CCP-negative patients. Anti-CCP seropositivity was also significantly associated with radiological changes, manifested by peripheral deformities and boney erosions. Similar results have been widely reported in RA, and anti-CCP seropositivity has been shown to predict the future development of joint destruction and disease progression [9-11].

In comparison with the previously published studies on anti-CCP and PsA, our results agreed with some of these studies [14-18], where anti-CCP seropositivity was associated with both clinical and radiological features. Other studies on the other hand, revealed associations of anti-CCP seropositivity only with polyarthritis [12,13]. Alenius et al. [12] reported no associations with deformities, functional impairment of peripheral joints or radiological changes.

Based on the results obtained in our study, the key question is whether these anti-CCP-positive patients truly have PsA or whether in fact they represent patients who actually have RA and psoriasis especially those with positive RF and symmetric polyarthritis. The differentiation between PsA and RA in patients with psoriasis is difficult [2]. The Moll and Wright criteria [5] excluded RF positive patients from a diagnosis of PsA. However, a false positive RF was found in as many as 13% of patients with psoriasis and in PsA as well [19]. Furthermore, RF seropositivity was found in HCV related conditions [22] and in elderly patients [23]. Regarding the 2 PsA patients with positive anti-CCP and RF, one had 39 years and the second had 45 years and they were HCV-negative. Using a higher cut-off value for RF may increase specificity of RF testing and reduce the number of false positive results. Further studies are however needed to clarify this suggestion.

Different reasons to favour PsA as the most likely diagnosis for these anti-CCP-positive patients were however present. First, none of the patients fulfilled the ARA criteria for RA. Second, all had arthritis and deformities of the distal interphalangeal joints. Lastly, 4 out of the 7 patients had psoriatic nail changes. The presence of higher number of involved, swollen and tender peripheral small joints and the associations with polyarthritis, functional impairment and deformities of peripheral joints and erosive arthritis may reflect the worse prognosis associated with anti-CCP in PsA as the case in anti-CCP-positive RA.

Many authors [24,25] demonstrated the presence of anti-CCP years before the clinical manifestation of RA. Therefore, another hypothesis could be that anti-CCP-positive PsA patients may suffer from an overlap with a preclinical form of RA and in such cases anti-CCP testing help in selection of patients who may need follow up, especially with ARA criteria. Our study is expanded to clarify this hypothesis, before putting a firm conclusion.

Even if the definite diagnosis remained in doubt, the evidence presented here suggests that as in RA, any patient with PsA and positive anti-CCP falls into a special category. Anti-CCP may be a marker for, or contribute to disease severity. It may be also one factor that influences treatment selection and response. Early treatment with disease modifying anti-rheumatic drugs (DMARD) whether conventional drugs as methotrexate or biological agents may prevent the progression of joint damage since no available treatment can reverse significant joint damage [26]. Prospective studies are warranted in this clinical setting. In RA, anti-CD20 monoclonal antibody (rituximab) has been shown to reduce circulating levels of anti-CCP [27].

## Conclusion

Although the number of anti-CCP-positive PsA in the present study was relatively small, yet the significant differences found in its serum concentrations between the studied groups help us to draw conclusions. Anti-CCP-seropositivity in PsA is associated with several clinical and radiological features and therefore, it may be considered a marker of, or contributes to disease severity. However, this study has to be expanded and focused on a large group of PsA to confirm any true association or future evolution of anti-CCP-positive patients to RA, before putting firm conclusions.

## Competing interests

The authors declare that they have no competing interests.

## Authors' contributions

NSA carried out the study design, participated in the recruitment of patients and controls, performed the dermatological examination and participated in the acquisition, analysis and interpretation of data, performed the statistical analysis, wrote and edited the manuscript. HEH participated in the study design, in the recruitment of patients and controls, performed the examination of joints and participated in the acquisition, analysis and interpretation of data. ZAG participated in the assessment of the laboratory parameters. EEO participated in the revision and completion of the statistical analysis. All authors read and approved the manuscript.
